# Immunoparesis in newly diagnosed Multiple Myeloma patients: Effects on overall survival and progression free survival in the Danish population

**DOI:** 10.1371/journal.pone.0188988

**Published:** 2017-12-07

**Authors:** Rasmus Sørrig, Tobias W. Klausen, Morten Salomo, Annette J. Vangsted, Ulf Christian Frølund, Kristian T. Andersen, Anja Klostergaard, Carsten Helleberg, Robert S. Pedersen, Per T. Pedersen, Sissel Helm-Petersen, Elena Manuela Teodorescu, Birgitte Preiss, Niels Abildgaard, Peter Gimsing, for the Danish Myeloma Study Group

**Affiliations:** 1 Department of Hematology, Rigshospitalet, Copenhagen, Denmark; 2 Department of Clinical Medicine, Faculty of Health and Medical Sciences, University of Copenhagen, Copenhagen, Denmark; 3 Hematological Research Laboratory, Herlev Hospital, Herlev, Denmark; 4 Department of Hematology, Zealand University Hospital, Roskilde, Denmark; 5 Department of Internal Medicine, Hematological section, Vejle Hospital, Vejle, Denmark; 6 Department of Hematology, Aarhus University Hospital, Aarhus, Denmark; 7 Department of Hematology, Herlev Hospital, Herlev, Denmark; 8 Department of Internal Medicine, Hematological section, Holstebro Hospital, Holsterbro, Denmark; 9 Department of Hematology, Esbjerg Hospital, Esbjerg, Denmark; 10 Department of Hematology, Aalborg University Hospital, Aalborg, Denmark; 11 Department of Pathology, Odense University Hospital, Odense, Denmark; 12 Department of Hematology, Odense University Hospital, Odense, Denmark; University of North Carolina at Chapel Hill, UNITED STATES

## Abstract

Immunoparesis (hypogammaglobulinemia) is associated to an unfavorable prognosis in newly diagnosed Multiple myeloma (MM) patients. However, this finding has not been validated in an unselected population-based cohort. We analyzed 2558 newly diagnosed MM patients in the Danish Multiple Myeloma Registry representing the entire MM population in Denmark from 2005–2013. Two-thousand two hundred and fifty three patients (90%) presented with reduction below lower normal levels of at least one uninvolved immunoglobulin. Using multivariable Cox regression we found that high age, high ISS score, high LDH and IgA MM were associated to both shorter overall survival and progression free survival. Furthermore, bone marrow plasma cell % was associated to short progression free survival. Immunoparesis had no independent significant effect on OS (HR 0.9 (95%CI: 0.7;1.0; p = 0.12)). Likewise, the number of suppressed immunoglobulins or the relative degree of suppressed uninvolved immunoglobulins from lower normal level (quantitative immunoparesis) was not associated to OS in the multivariable analysis. However, quantitative immunoparesis with at least 25% reduction (from lower normal level) of uninvolved immunoglobulins was associated to shorter PFS for the entire population. The impact of quantitative immunoparesis on PFS was present irrespective of calendar periods 2005–2008 and 2009–2013. Our population-based study does not confirm that immunoparesis at diagnosis is an independent prognostic factor regarding OS. However, quantitative immunoparesis is associated to a shorter PFS.

## Introduction

Outcome for patients with Multiple Myeloma (MM) is highly variable, which can be related to specific tumor characteristics, host factors, tumor burden and disease complications[1]. The International Staging System (ISS) and classification of chromosomal abnormalities are accepted standards for prognostication in MM and a combination of ISS and FISH abnormalities with plasma LDH levels has recently proven to be a reliable prognostic tool for assessing survival outcome of newly diagnosed MM patients enrolled in protocols[2–4].

At diagnosis the majority of patients with MM presents with suppression of one or more of the uninvolved immunoglobulins (immunoparesis)[5]. The pathogenesis behind the suppression of the polyclonal immunoglobulin production from normal plasma cells (PC) is complex and studies have reported immune impairment caused by dysregulation of both the normal T–and B-cell repertoire and of soluble B-cell maturation agent (BCMA) in MM patients[6–10]. In Monoclonal Gammopathy of Undetermined Significance (MGUS) and Smoldering Multiple Myeloma (SMM), immunoparesis has been shown to be a prognostic marker for progression to symptomatic MM. In a Spanish cohort immunoparesis combined with aberrant PC immune phenotype could predict risk of progression in patients with MGUS and SMM[11]. Our group has recently confirmed that immunoparesis is an important independent risk factor for progression from SMM to MM and immunoparesis can together with the M-protein level stratify patients into 3 risk categories[12]. In symptomatic MM, a Greek multicenter study has shown that patients with immunoparesis at diagnosis have a significant worse overall survival (OS) compared to patients with normal uninvolved immunoglobulins[13]. In a subgroup of the Greek cohort, they found that presence of immunoparesis also shortened the progression free survival (PFS). However, these findings have not been validated in a population-based setting. In addition, it is unknown if the prognostic impact is the same for patients treated with newer anti-myeloma regimens including immunomodulary drugs (IMIDs) and proteasome inhibitors.

The primary aim of this study was to evaluate the independent prognostic importance of immunoparesis for OS and PFS in a large population-based cohort using the Danish Multiple Myeloma Registry (DMMR), which includes information on all MM patients in Denmark diagnosed since 2005.

## Materials and methods

### Study population

We analyzed data of 2558 newly diagnosed MM patients in the DMMR from 1 January 2005 to 31 December 2013. All patients but one (total cohort n = 2557) had complete follow up data for survival and time to first relapse. The DMMR contains data on baseline biochemistry (including immunoglobulin levels by standard nephelometry or tubidimetry), treatment regimens and response to treatment. The content of DMMR has been described in detail previously[12]. All MM diagnosed patients in DMMR are compared with the National Patient Registry in Denmark which ensures that no patients are missed[14]. A recent validation of the DMMR has shown that data are of high quality[15]. During the study period high-dose melphalan with autologous stem cell transplantation (ASCT) was recommended for all transplant-eligible patients <65 years of age and optional for fit patients ≥ 65 years. The induction regimens have changed substantially during the study period. From 2005–2009 the standard induction regimens for transplant-eligible patients outside clinical trials was based on combinations of cyclophosphamide-dexamethasone (CyDex). A minority of patients were treated with vincristine, doxorubicin, and dexamethasone (VAD)[16]. The elderly or transplant-ineligible MM patients were treated with melphalan-prednisolone (MP) or MP + thalidomide (MPT) during 2005–2009. In 2009 bortezomib in combination with dexamethasone or cyclophosphamide-dexamethasone (VD or VCD) was introduced as first line standard induction for transplant-eligible patients in Denmark[16]. For transplant ineligible patients the first line treatment from 2009–2013 was based on MPT or MPV or lenalidomide-dexamethasone (Len-Dex). All patients met the 2003 International Myeloma Working Group (IMWG) criteria for diagnosis[17]. Progressive disease was defined according to 2006 IMWG criteria[18]. Immunoparesis was defined qualitatively as one or more of uninvolved immunoglobulins below the normal levels IgG < 6.1 g/L, IgA < 0.70 g/L and/or IgM <0.39g/L at time of diagnosis, as described previously[12]. Quantitative immunoparesis was defined as a gradual 25–75% reduction in uninvolved immunoglobulins from lower normal level. All the biochemical analyses were performed by local clinical biochemical laboratories at the different hematological sites. All Danish laboratories are accredited to international standards according to General Requirements for the Competence of Testing and Calibration Laboratories (ISO/IEC 17025:2005).

### Statistics

Baseline patient characteristics were described using median, interquartile range (IQR) and frequencies and percentages for continuous values. Mann–Whitney *U* test, X^2^ test and Fisher’s exact test were used to compare differences between patient characteristics when appropriate. Cox proportional hazard regression analysis was used to estimate risk factors for overall survival (OS) and progression free survival (PFS) with hazard ratios (HR) and 95% confidence intervals (CI). We performed a multivariable Cox regression including immunoparesis and univariable significant risk factors. We used backward selection to show HR and p-values for all risk factors after the exclusion of non-significant variables. Survival curves were estimated using the Kaplan-Meier method.

Statistical analyses were performed using SPSS software version 22.0 (IBM corporation, Armonk NY, USA) and R version 3.2.3 (R Foundation for Statistical Computing, Vienna, Austria). This study was approved by the Danish Data Protection Agency (J.no. 2012-58-0004 and 30–1269) and the Danish Patient Safety Authority (J.no. 3-3013-676/1).

## Results

Median follow-up for OS was 77 months and 61 months for PFS. Median age at diagnosis was 70 years and the female to male ratio was 0.45 (Table 1). Median bone marrow plasma cell percentage (BMPC%) was 31%. Fifty-four percent presented with IgG M-protein, 21% IgA, 15% with Light Chain Disease (LCD) only and the median M-protein at diagnosis was 2.8 g/dL.

**Table 1:** Baseline characteristics for the entire cohort.

	MM (n = 2557)	-Immunoparesis (n = 254)	+immunoparesis (n = 2253)	P
**Age**	70 (62;77)[30;98]	70 (63;77)[30;93]	70 (62;77)[32;98]	0.89
**Gender (F/M)**	1147 (45%) / 1410 (55%)	84 (33%) / 170 (67%)	1042 (46%)/1211 (54%)	<0.0001
**BMPC% (n = 2471)**	31 (16;51)[0;100]	14 (8;25)[0;100]	35 (20;55)[0;100]	<0.0001
**P-M-Protein**				0.09
**IgA**	525 (21%)	48 (19%)	472 (21%)	
**IgG**	1376 (54%)	145 (57%)	1211 (54%)	
**LCD**	388 (15%)	29 (11%)	355 (16%)	
**IgM**	12 (<1%)	3 (1%)	8 (<1%)	
**IgD**	17(<1%)	1 (<1%)	16 (2%)	
**IgE**	2(<1%)	0 (0%)	2 (<1%)	
**More than one**	104 (4%)	12 (5%)	92 (4%)	
**Non-secretory**	53 (2%)	9 (4%)	43 (2%)	
**Unknown**	80 (3%)	7 (3%)	54 (2%)	
**M-Protein konc. (g/dL) n = 2171**	2.8(1.2;4.5)[0;17.2]	1.6(0.6;2.9)[0;6.9]	3.0(1.3;4.7)[0;17.2]	<0.0001
**Urine M-protein**				0.019
**Unknown**	765 (30%)	68 (27%)	668 (30%)	
**No**	623 (24%)	81 (32%)	535 (24%)	
**Yes**	1169 (46%)	105 (41%)	1050 (46%)	
**If Yes**:				
**Kappa**	712 (61%)	65 (62%)	640 (61%)	0.85
**Lambda**	422 (36%)	36 (34%)	379 (36%)	0.71
**Uspecified**	52 (4%)	5 (5%)	47 (4%)	0.88
**Immunoparesis (Yes/No)** **(50 unknown)**	2253 (90%) / 254 (10%)	-	-	-
**Immunoparesis no of Ig**				
**0**	10%(254)			
**1**	19% (460)			
**≥2**	71%(1734)			
**Immunoparesis (25–49% below lower normal level)**	81% (2067)			
**Immunoparesis (50–74% below lower normal level)**	67% (1673)			
**Immunoparesis (75% or more below lower normal level)**	32% (800)			
**Beta-2-Microglobulin (mg/l)** **n = 2126**	4.3 (2.7;6.9)[0;40.8]	2.7 (2.0;4.7)[0.7;30.3]	4.4 (2.8;7.1)[0;40.8]	<0.0001
**Albumin (g/l)** **n = 2433**	36 (31;40)[9;100]	39 (33;42)[16;51]	36 (31;40)[9;100]	<0.0001
**Creatinine (μmol/l) n = 2521**	91 (71;141) [30;2000]	85 (70;111)[30;740]	93 (71;146)[33;2000]	0.0005
**LDH (U/l) n = 2444**	174 (142;220)[0;1683]	171 (145;205)[0;1368]	174 (142;221)[0;1683]	0.53
**ISS**				<0.0001
**I**	582 (28%)	116 (55%)	465 (25%)	
**II**	760 (36%)	51 (24%)	701 (38%)	
**III**	752 (36%)	45 (21%)	698 (37%)	
**Unknown**	463	42	389	
**FISH analyses (n = 417)**^a^	417 (16%)	42 (17%)	372 (17%)	
**t(4,14)**		5 (2%)	63 (3%)	
**t(14,16)**		2 (<1%)	15 (<1%)	
**del 17p**		2(<1%)	27 (1%)	
**First line treatment**				
**Thalidomide**^b^	519 (20%)	36 (14%)	472 (21%)	
**Lenalidomide**^b^	131 (5%)	10 (4%)	120 (5%)	
**Bortezomib**^b^	769 (30%)	61 (24%)	702 (31%)	
**ASCT completed**	721 (28%)	63 (25%)	647 (28%)	
**≤65 years**	615 (85%)			
**>65 years**	106 (6%)			
**Allo-HST**	4 (<1%)	0	4	
**Cytostatics**^b^	2087 (82%)	193 (76%)	1860 (83%)	
**MP**^b^	875(45%)	92 (36%)	768 (34%)	
**VAD**^b^	31 (2%)	1 (<1%)	29 (1%)	
**CyDex**^b^	671 (34%)	55 (22%)	604 (27%)	
**Other cytostatic unspecified**	382 (20%)			
**Year of diagnosis**				0.14
**2005–2008**	1097 (43%)	119 (47%)	943 (42%)	
**2009–2013**	1460 (57%)	135 (53%)	1310 (58%)	
**Response to first line treatment**				
**≥ CR**	436 (17%)	43 (17%)	391 (17%)	0.87
**≥ PR**	1489 (60%)	138 (58%)	1331 (61%)	0.45

One patient was excluded from the study due to lack of follow-up data for PFS and OS. P-values indicate tests for comparison of differences between patients with and without immunoparesis (see text for details). Interquartile range (IQR) for continuous variables is shown in brackets and range in solid brackets. Percentages (%) for categorical variables are shown in brackets. Immunoparesis = one or more of uninvolved immunoglobulins below the lower normal levels IgG < 6.1 g/L, IgA < 0.70 g/L and/or IgM <0.39g/L. BMPC% = bone marrow plasma cell %, ASCT = High-dose Melphalan with autologous stem cell transplantation. Allo-HST = allogeneic stem cell transplantation, MP = Melphalan-prednisone, VAD = vincristine, doxorubicin, and dexamethasone, Cy-dex = Cyclophosphamide-dexamethasone. CR = Complete Response, PR = Partial Response.

^a^FISH (Fluorescence in situ hybridization). Only FISH results positive for the adverse abnormalities; t(4;14), t(14;16) and deletion17p (del17p).

^b^Percentages of specified treatment is related to the total number of patients in each column; n = 2557, n = 254 and n = 2253 respectively. The percentages do not add up to 100% in each column as patients might have received more than one of the specified treatments.

Ninety % presented with immunoparesis of at least one uninvolved immunoglobulin below normal level; 19% of which had reduction in one uninvolved immunoglobulin and 71% with a reduction in 2 or more of the uninvolved immunoglobulins. Eighty-one percent of patients with immunoparesis had at least a 25% reduction in immunoglobulin level from lower normal limit, 67% with at least 50% reduction and 32% with at least a 75% reduction (Table 1). In the 254 patients without immunoparesis 29 patients (11%) had LCD myeloma.

There was no significant age difference between patients with normal uninvolved immunoglobulin levels and patients with immunoparesis (Table 1). Relatively more female than male patients presented with immunoparesis (91% vs. 86% respectively). Both baseline median BMPC% and M-protein level was significantly higher in patients with immunoparesis (BMPC% IQR 35(20;55) vs. IQR 14(8;25); p<0.0001, and M-protein 3.0g/dL IQR (1.3;4.7) vs. 1.6g/dL IQR(0.6;2.9); p<0.0001). Furthermore, more patients with immunoparesis were staged ISS score II and III compared to patients without immunoparesis (38% (ISSII) and 37%(ISSIII) vs. 24%(ISSII) and 21% (ISSIII)). No relevant difference in the prevalence of immunoparesis was seen among patients treated with ASCT or without ASCT (28% vs. 25%) (Table 1). There were more patients with immunoparesis who received IMiDs and Bortezomib in first line treatment compared to patients with no immunoparesis (for Thalidomide 21% vs. 14%, Bortezomib 31% vs. 24%). We found no significant difference between the number of patients with immunoparesis who achieved Complete Response (CR) or Partial response (PR) (to first line treatment) compared to patients without immunoparesis (Table 1: p = 0.87 and p = 0.45, respectively).

Fluorescence In Situ Hybridization (FISH) was introduced for MM diagnostic workup in a minority of centers in Denmark prior to 2008 and has not systematically been reported to the DMMR during the study period. Therefore, FISH data was only available in 16% (n = 417) of cases (Table 1).

### Prognostic factors for overall survival and progression free survival in Danish Multiple Myeloma patients

**Fig 1:**
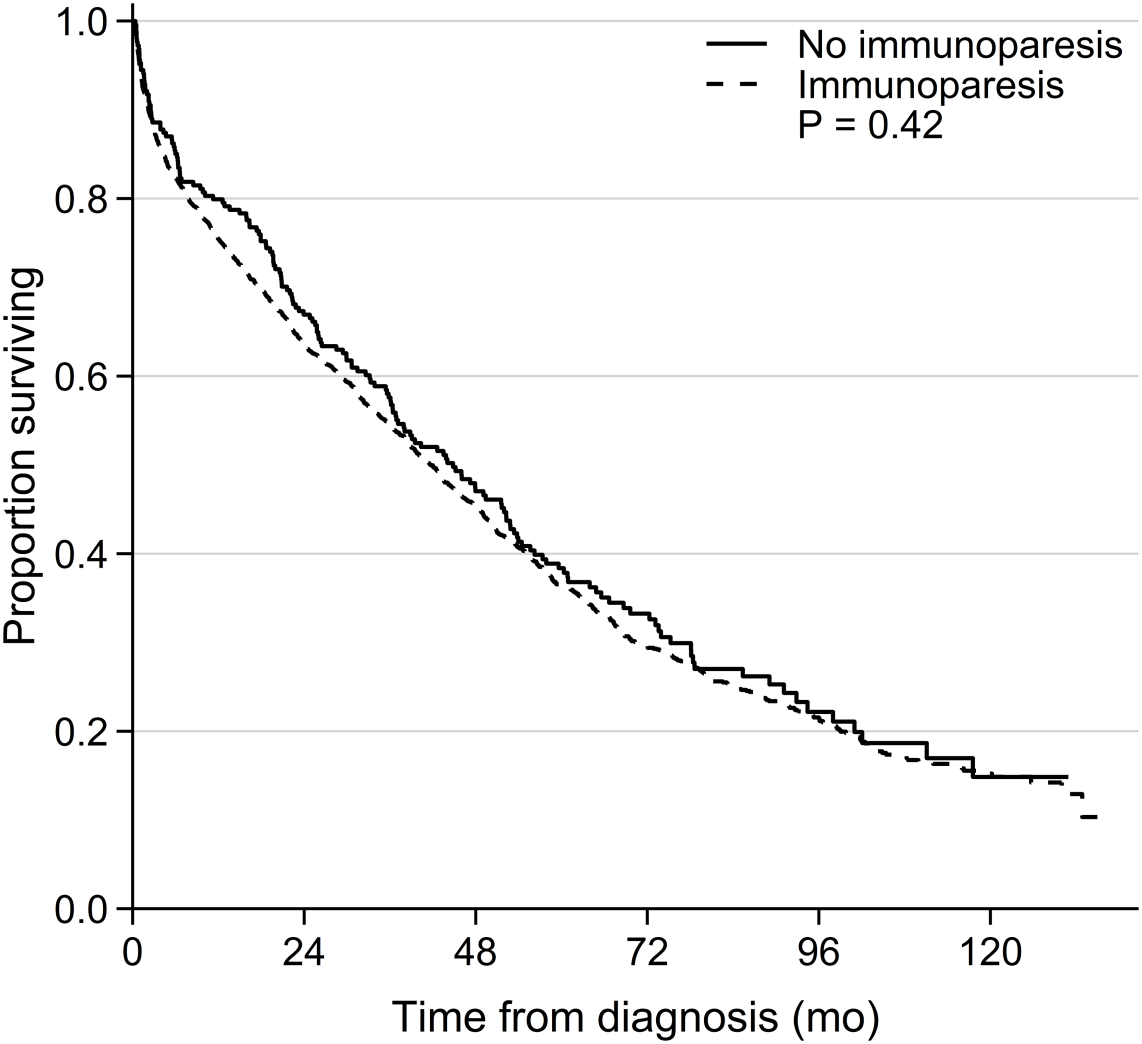
OS for patients with and without qualitative immunoparesis.

**Fig 2:**
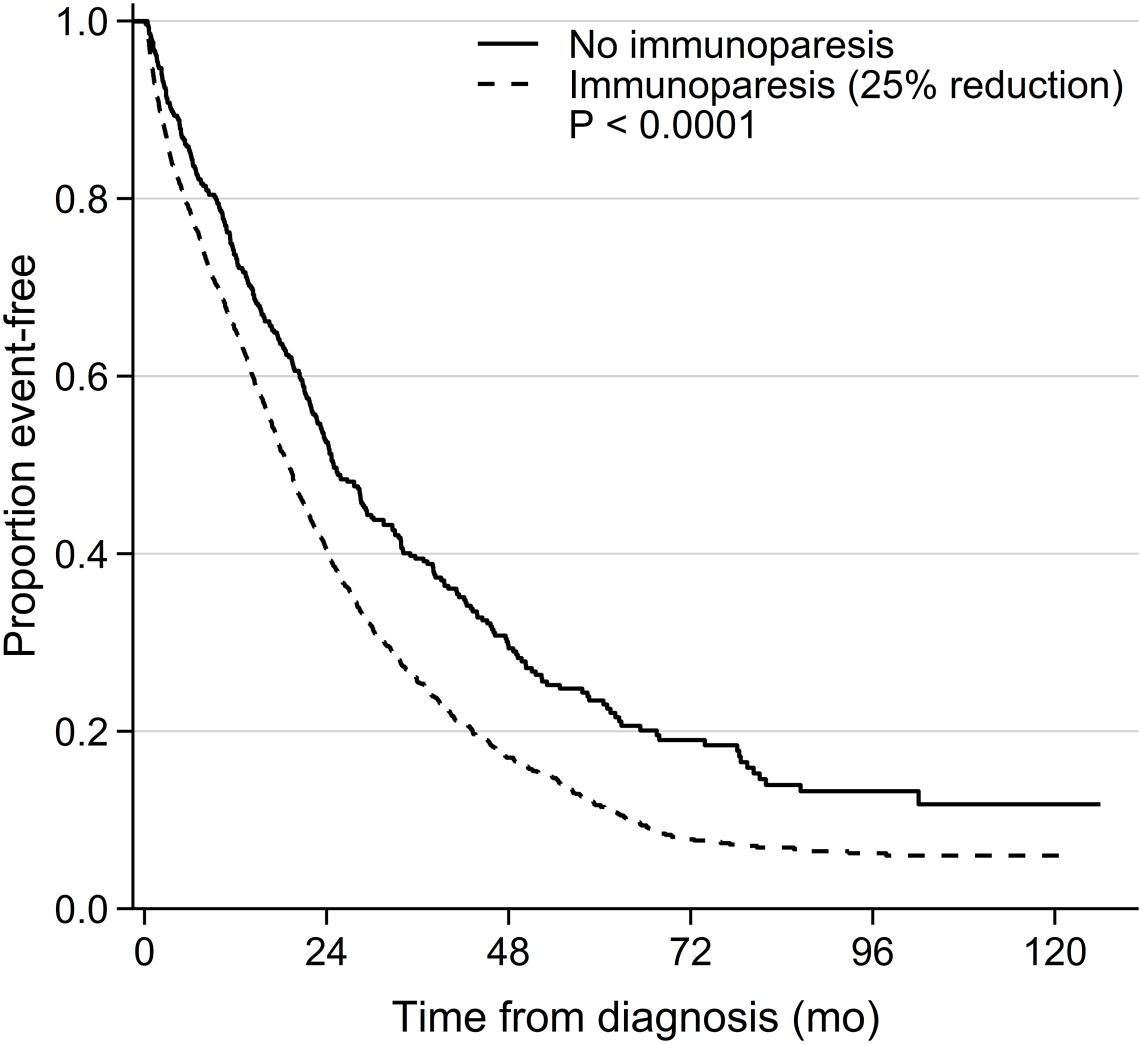
PFS for patients with quantitative immunoparesis of 25% (from lower normal level) compared to patients without immunoparesis.

A univariable analysis of potential risk factors for OS and PFS was performed for all patients in the cohort (all data are available in S1 Table). As expected high age (>65 years), increasing BMPC%, ISS score, creatinine, LDH levels and IgA M-protein significantly reduced the OS. Quantitative immunoparesis of 25% or 50% reduction (from lower normal level) in uninvolved immunoglobulin levels was associated to a significant poorer OS (S1 Table). However, qualitative immunoparesis was not significantly associated to OS in the univariable analysis (S1 Table, Fig 1). For PFS univariable significant risk factors were high ISS score, creatinine, LDH and M-protein levels. Both qualitative and quantitative immunoparesis was associated to a shorter PFS in the univariable analysis (S1 Table, Fig 2). Furthermore, female patients with a quantitative immunoparesis of 25% reduction (from lower normal level) in uninvolved immunoglobulin were significantly associated to worse OS and PFS (S1 Table).

### Independent risk factors for overall survival and progression free survival

Significant risk factors for both OS and PFS in the univariable analysis were included in a multivariable Cox regression analyses (Tables 2–4). Age, IgA M-protein, ISS score and high LDH were risk factors for OS in the multivariable analysis (p<0.0001), whereas increasing BMPC% failed as an independent risk factor for OS (Table 2). Qualitative immunoparesis was not a significant risk factor for OS in the multivariable analysis (HR 0.9 (95%CI: 0.7;1.0; p = 0.12).

**Table 2:** Multivariable analysis of factors for OS.

	Backward selection^a^ (N = 1996)	
	HR (95% CI)	P value
**Age >65**	2.3 (2.0;2.6)	<0.0001
**ISS**		<0.0001
**I**		
**II**	1.7 (1.4;1.9)	<0.0001
**III**	2.3 (2.0;2.6)	<0.0001
**Creatinine (above normal)**	-	-
**LDH (above normal)**	1.4 (1.2;1.6)	<0.0001
**BMPC% (cont.)**	-	-
**IgA MM**	1.3 (1.2;1.5)	<0.0001
**Immunoparesis**	-	-

**Table 3:** Multivariable analysis of factors for PFS.

	Backward selection^a^ (N = 1885)	
	HR (95% CI)	P value
**Age >65**	1.6 (1.5;1.8)	<0.0001
**ISS**		<0.0001
**I**		
**II**	1.4 (1.2; 1.6)	<0.0001
**III**	1.7 (1.5;1.9)	<0.0001
**Creatinine (above normal)**	-	-
**LDH (above normal)**	1.2 (1.0;1.3)	0.018
**BMPC% (cont.)**	1.1 (1.0;1.1)	0.011
**IgA MM**	1.2 (1.1;1.4)	0.005
**M-protein (<3, ≥3)**	-	-
**Immunoparesis**	-	-

**Table 4:** Multivariable analysis of both qualitative and quantitative immunoparesis as risk factors for OS and PFS in all patients.

All patients	OS^a^ HR (95% CI)	OS P	PFS^a^ HR (95% CI)	PFS P
**Immunoparesis (0 ref,1 vs. 2 Ig)**				
**0**	1	*0*.*16*	1	0.023
**1**	0.8 (0.7; 1.0)	*0*.*058*	1.0 (0.8; 1.3)	0.71
**2**	0.9 (0.7; 1.1)	*0*.*24*	1.3 (1.0; 1.5)	0.018
**Immunoparesis (25–49% below lower normal level)**	1.0 (0.9; 1.2)	*0*.*56*	1.3 (1.2; 1.6)	<0.0001
**Immunoparesis (50–74% below lower normal level)**	1.0 (0.9; 1.2)	*0*.*65*	1.2 (1.1; 1.4)	0.0007
**Immunoparesis(75% or more below lower normal level)**	0.9 (0.8; 1.1)	*0*.*35*	1.2 (1.0; 1.3)	0.010

^a^HR and p-values for OS and PFS after adjusting for all significant variables (age>65 years, ISS score, LDH levels, IgA MM for OS and age>65 years, ISS score, LDH, BMPC% and IgA MM for PFS) in a multivariable cox regression model.

LDH = Lactate dehydrogenase, BMPC% = Bone marrow plasma cell %. Cont. = continuous value. Immunoparesis = one or more of uninvolved immunoglobulins below the lower normal levels IgG < 6.1 g/L, IgA < 0.70 g/L and/or IgM <0.39g/L. Immunoparesis 1 or 2 = one or two of uninvolved immunoglobulins below the lower normal levels. Immunoparesis 25%, 50% and 75% red = 25% or 50% or 75% reductions of uninvolved immunoglobulins below the lower normal level.

Using the same multivariable model, we found that high age and ISS score at diagnosis were significant independent risk factors for PFS (p<0.0001) (Table 3). High baseline LDH, high BMPC% and IgA MM showed a modest albeit significant association to shorter PFS (HR 1.2(95%CI: 1.0;1.3; p = 0.018), HR1.1 (95%CI: 1.0;1.1; p = 0.011) and HR 1.2 (1.1;1.4); p = 0.005 respectively). Furthermore, both qualitative and quantitative immunoparesis was tested for impact on OS and PFS when adjusting for significant risk factors in the multivariable analysis (Table 4): We found no significant association of either quantitative or qualitative immunoparesis to a shorter OS. Qualitative immunoparesis was near significant risk factor for PFS (HR 1.2 (95%CI: 1.0; 1.4; p = 0.054)). However, quantitative immunoparesis with either 25%, 50% or 75% reduction in uninvolved immunoglobulin levels was significant after adjusting for all independent risk factors (HR 1.3 (95%CI: 1.2; 1.6; p<0.0001), HR 1.2 (95%CI: 1.1; 1.4; p = 0.007) and HR 1.2 (95%CI: 1.0; 1.3; p = 0.010) respectively). We also tested the impact of immunoparesis on OS and PFS in different age groups (Table 5). Patients ≤65 years of age (n = 890) with qualitative immunoparesis had a better survival compared to patients with no immunoparesis when adjusting for significant prognostic markers (HR 0.7 (95%CI: 0.5;0,9; p = 0.017), Table 5). The survival advantage was not present in patients with a gradual 25% to 75% decrease in uninvolved immunoglobulin levels. Neither qualitative nor quantitative immunoparesis was associated to a shorter OS of the older population >65 years (n = 1667) (Table 5). However, immunoparesis in two uninvolved immunoglobulins and a 25% or 50% reduction in uninvolved immunoglobulin levels remained significant factors for a shorter PFS for older patients (>65 years) (HR 1.3 (95%CI: 1.0;1.7; p = 0.024), HR 1.4 (95%CI: 1.2;1.7; p = 0.0002) and HR 1.2 (95%CI: 1.1;1.4; p = 0.005), respectively (Table 5)).

**Table 5:** Immunoparesis and association to OS and PFS in different age groups.

**≤ 65 (n = 890)**	**OS**		**PFS**	
	**HR (95% CI)**	**P**	**HR (95% CI)**	**P**
**Immunoparesis (Yes <> no)**	0.7 (0.5; 0.9)	0.017	1.1 (0.8; 1.6)	0.41
**Immunoparesis (0 ref,1 vs. 2 Ig)**				
**0**	1	0.064	1	0.69
**1**	0.7 (0.5; 1.0)	0.072	1.2 (0.8; 1.7)	0.41
**2**	0.6 (0.5; 0.9)	0.014	1.1 (0.8; 1.6)	0.44
**Immunoparesis (25% below lower normal level)**	0.9 (0.7; 1.2)	0.49	1.2 (0.9; 1.6)	0.13
**Immunoparesis (50% below lower normal level)**	0.9 (0.8; 1.2)	0.62	1.2 (1.0; 1.5)	0.061
**Immunoparesis (75% below lower normal level)**	0.9 (0.7; 1.1)	0.37	1.2 (1.0; 1.4)	0.051
**> 65 (n = 1667)**	**OS**		**PFS**	
	**HR (95% CI)**	**P**	**HR (95% CI)**	**P**
**Immunoparesis (Yes <> no)**	1.0 (0.8; 1.2)	0.77	1.2 (1.0; 1.5)	0.10
**Immunoparesis (0 ref,1 vs. 2 Ig)**				
**0**	1	0.16	1	0.0007
**1**	0.8 (0.7; 1.1)	0.22	1.0 (0.7; 1.3)	0.86
**2**	1.0 (0.8; 1.3)	0.98	1.3 (1.0; 1.7)	0.024
**Immunoparesis (25% below lower normal level)**	1.1 (0.9; 1.3)	0.25	1.4 (1.2; 1.7)	0.0002
**Immunoparesis (50% below lower normal level)**	1.1 (0.9; 1.2)	0.38	1.2 (1.1; 1.4)	0.005
**Immunoparesis (75% below lower normal level)**	1.0 (0.8; 1.1)	0.60	1.1 (1.0; 1.3)	0.092

HR and p-values represents values when adjusting for independent risk factors for OS and PFS shown in Tables 2 and 3. Immunoparesis = one or more of uninvolved immunoglobulins below the reference levels IgG < 6.1 g/L, IgA < 0.70 g/L and/or IgM <0.39g/L. Immunoparesis 1 vs. 2 Ig = 1 or 2 uninvolved immunoglobulins below reference level.

Bortezomib was introduced in standard induction regimens in Denmark in 2009. We analyzed if there was a difference in the effects of immunoparesis on OS and PFS in patients diagnosed from 2005–2008 (n = 804) and 2009–2013 (n = 1183) (S2 Table). For patients >65 years we found that 25% reduction in uninvolved immunoglobulin was significantly associated to a shorter PFS irrespective of calendar period 2005–2008 or 2009–2013 (HR 1.4 (95%CI: 1.1;1.9;p = 0.019) and HR 1.4 (95%CI: 1.1;1.8; p = 0.007) respectively). For patients <65 years we observed no significant impact on PFS in the different calendar periods.

## Discussion

This is the first population-based retrospective cohort study of immunoparesis as an independent risk factor for OS and PFS in symptomatic multiple myeloma. A multivariable analysis showed that high age, ISS score, high LDH and IgA subtype were associated to shorter OS and PFS. Furthermore, high BMPC% was associated to shorter PFS. Surprisingly, immunoparesis defined qualitatively as one or more uninvolved immunoglobulins below lower normal level or quantitatively had no significant effect on OS when adjusting for all other significant prognostic markers available in our cohort (Table 2 and Fig 1). Importantly, we found that quantitative immunoparesis (with at least 25%, 50% or 75% immunoglobulin reduction) remained a significant independent risk factor for PFS in the entire population in a multivariable analysis (Table 4 and Fig 2).

Our results regarding OS are in disagreement with the findings in a large Greek multicenter study[13]. Here the authors found that patients with no immunoparesis had significantly improved survival when adjusting for other prognostic factors. The survival benefit for patients without immunoparesis was 55 months vs. 41.5 months for patients with immunoparesis[13]. In a subgroup analysis from a single center the authors also showed that immunoparesis was an independent marker for shorter PFS[13]. Another retrospective study showed improved OS and PFS in patients without immunoparesis at diagnosis in patients treated in Medical Research Council (MRC) MM clinical trials from 1980–1997[19]. There are important differences between our study, the Greek study and the MRC trials. The incidence of immunoparesis in the Greek study is comparable to our study (87% vs. 90%), however, in the Greek study patients were included over a 22 year (1990–2012) period thus before and after the introduction of novel anti-myeloma drugs. The authors found that immunoparesis was associated to a poorer OS in a multivariable analysis including treatment with IMIDs or Bortezomib upfront[13]. However, since only 38% of patients in the Greek study received IMIDs or Bortezomib the multivariable analysis could only have been performed in a subset of their cohort, which is not specified by the authors[13]. Also, it is not clear how many patients where included in the most recent calendar period of their study where novel agents may have been used in both first line treatment and in the relapse setting. Furthermore, novel agents were not shown to be independent markers for PFS in the multivariable analysis including immunoparesis[13]. The MRC study was based on survival data from studies before the introduction of novel agents, and patients had arguably a different outcome in OS and PFS than our cohort[19]. Furthermore, it is not clear whether the prevalence of immunoparesis in the MRC data is comparable to our study and the Greek study[19]. In a recent Turkish single-center study of 137 newly diagnosed MM patients from 2003–2015, the prognostic importance of immunoparesis at diagnosis has been analyzed[20]. In line with our study, the authors could not find a significant association of immunoparesis with OS in patients treated with novel regimens[20]. Our multivariable analysis included all significant univariable risk factors for both PFS and OS with a limited degree of missing data. Furthermore, to our knowledge, our study is the first to investigate the impact of both qualitative and quantitative immunoparesis in a multivariable analysis and we found no association to poorer OS for either of the definitions. We therefore do not find evidence that immunoparesis represents a proxy for disease burden nor serve a marker for aggressive MM. Age was the most influential risk factor for both OS and PFS in our cohort. We therefore analyzed if there were major differences in the prognostic effect of immunoparesis in ages below 65 and above 65 years. For the younger population (≤65 years) we found that immunoparesis was actually associated to a slightly better survival when adjusted for all significant risk factors (Table 5). This small survival benefit seems paradoxical as we would expect patients with immunoparesis to be more prone to infections which has been described as one of the most important risk factors for early death irrespective of patient age[21]. We do not have data on infections, causes of death nor complete information to assess other prognostic features such as high-risk FISH, which could explain this finding. In the older population (>65 years) there was no significant effect of immunoparesis on OS (Table 5). In a previous study of transplant in-eligible MM patients in Denmark (the vast majority being >65 years old) our group found that infections are the leading cause of early deaths [22]. Significantly more patients with immunoparesis died within the first 180 days compaired to patients alive after 180 days[22]. However, immunoparesis was not evaluated as an independent risk factor for infections [22].

Most studies define immunoparesis in MM qualitatively; as *any* of the uninvolved immunoglobulins below the lower normal level[13,23–25]. Using this definition, we could only find a trend towards immunoparesis as a predictor of a short PFS. However, a quantitative suppression in uninvolved immunoglobulins had a significant impact on PFS, also after adjusting for other significant variables. A significant increased HR for poorer PFS was found when patients had a reduction in 2 uninvolved immunoglobulins.

Furthermore, we found the 25% reduction in uninvolved immunoglobulin to be significantly associated to shorter PFS for patients > 65 years after adjusting for calendar periods both before and after 2009 where major changes in induction regimens were made (S2 Table). Interestingly, female MM patients with at least 25% immunoglobulin reduction (but not minor reductions in normal immunoglobulins) were associated to adverse OS and PFS in the univariable analysis (S2 Table). Whether a difference in reference interval for healthy men and women [26] can influence the rate of immunoparesis among male and female myeloma patients is unknown. A shorter PFS in patients with immunoparesis might be attributed to the changes in the distribution of normal vs. clonal plasma cells in the BM. Paiva et al. has shown that patients with >5% polyclonal PCs have a better PFS [27]. However, the authors did not analyze if there were a direct correlation of polyclonal PCs with the degree of immunoparesis. We argue that a clinical relevant level of immunoparesis for symptomatic MM should be at least 25% reduction in one or more uninvolved immunoglobulins.

There are several strengths to our study. In the Danish Health Care System all MM patients are diagnosed, treated and followed in public hospitals with hematological expertise. This ensures that our cohort consists of unselected patients and that data represents the entire Danish Multiple Myeloma population. The complete follow-up data in the DMMR provides a unique opportunity to evaluate the epidemiology and prognostic features of immunoparesis and other common MM characteristics in a population-based setting. All laboratories that provide data for the DMMR are regulated under the same international standards.

There are some limitations to our study. The treatment data registered in the DMMR refers to whether the patients at some point in the first line treatment received the given drug, but not in which specific treatment protocol the drug was used. Like other studies, we have no data on how many patients received immunoglobulin substitution therapy or whether this replacement therapy could influence the effects of immunoparesis at diagnosis. However, the treatment guidelines for newly diagnosed and relapse or refractory MM patients in Denmark are based on national consensus recommendations which are updated annually by the Danish Myeloma Study Group[28]. Immunoglobulin substitution therapy is generally not part of routine practice in Danish Myeloma centers regardless of immunoparesis status. Furthermore, risk stratification using FISH, was only available for a subgroup of our patients. Therefore, the prognostic effect of immunoparesis could not be evaluated independently of high-risk FISH status.

In conclusion our populations-based study shows that immunoparesis at diagnosis is not an independent prognostic factor regarding OS. The data suggests that immunoparesis with at least 25% reduction in uninvolved immunoglobulins at the time of diagnosis can identify patients with a shorter PFS. Further investigation of the consequences of immunoparesis and other secondary immune defects including the risk of infections, early deaths and/or progression in symptomatic Multiple Myeloma patients is needed.

## Supporting information

S1 Table A univariable analysis of potential risk factors for OS and PFS for all patients in the cohort. (PDF)

S2 Table Multivariable analysis of the effect of immunoparesis in calendar periods 2005–2008 and 2009–2013 divided by age groups. (PDF)
