# Supplementary material for: Immunoparesis in newly diagnosed Multiple Myeloma patients: Effects on overall survival and progression free survival in the Danish population
Source: PLoS One. 2017 Dec 7;12(12):e0188988. doi: 10.1371/journal.pone.0188988 (PMC5720701; doi:10.1371/journal.pone.0188988)
Supplement: S1 Table — (PDF) [file pone.0188988.s001.pdf]

S1 table: A univariable analysis of potential risk factors for OS and PFS for all patients in the cohort

MM n = 2557<br>OS , Median Follow up: 77 mo<br>PFS, median follow-up: 61 mo	OS<br>HR (95% CI)	OS<br>P value	PFS<br>HR (95% CI)	PFS<br>P value
**Age (≤65, >65)**	2.4 (2.2;2.7)	<0.0001	1.6 (1.5; 1.8)	<0.0001
**Age (<66, 66-80, >80)**		<0.0001		<0.0001
**<66**	1		1	
**66-80**	2.1 (1.9;2.3)	<0.0001	1.6 (1.4; 1.7)	<0.0001
**>80**	4.4 (3.9;5.1)	<0.0001	2.0 (1.8;2.3)	<0.0001
**Gender (Female ref)**	1.0 (0.9;1.1)	0.54	1.1 (1.0; 1.2)	0.15
**BMPC% (cont., HR pr 25%)**	1.1 (1.1;1.2)	<0.0001	1.1 (1.1;1.2)	<0.0001
**M-protein g/dL (<3, >=3)**	0.9 (0.9;1.1)	0.31	1.1 (1.0; 1.3)	0.005
**M-protein (cont., HR pr. 1 g/dL)**	0.98 (0.96;1.00)	0.069	1.03 (1.01;1.05)	0.001
**IgA M-protein**	1.3 (1.1;1.4)	<0.0001	1.1 (1.0;1.3)	0.027
**IgG M-protein**	0.8 (0.8;0.9)	0.0002	1.0 (0.9; 1.1)	0.52
**Immunoparesis (Yes <> no)**	1.1 (0.9;1.2)	0.42	1.3 (1.1; 1.6)	0.0002
**Immunoparesis (0 ref,1 vs. 2 Ig)**		0.38		<0.0001
**0**	1			
**1**	1.0 (0.8;1.2)	0.98	1.1 (0.9; 1.4)	0.18
**2**	1.1 (0.9;1.3)	0.37	1.4 (1.3; 1.6)	<0.0001
**Immunoparesis (25% below lower normal level)**	1.2 (1.1;1.4)	0.002	1.4 (1.3; 1.6)	<0.0001
**Immunoparesis (50% below lower normal level)**	1.1 (1.0;1.2)	0.043	1.3 (1.2; 1.4)	<0.0001
**Immunoparesis (75% below lower normal level)**	0.9 (0.9 ;1.0)	0.29	1.1 (1.0; 1.2)	0.009
**Immunoparesis (Yes <> no) (Females only)**	1.2 (0.9; 1.6)	0.17	1.4 (1.0; 1.8)	0.021
**Immunoparesis (25% red) (Females only)**	1.5 (1.2; 1.8)	0.0003	1.5 (1.2; 1.8)	<0.0001
**B2M (<3.5, >= 3.5)**	2.1 (1.9;2.3)	<0.0001	1.6 (1.5; 1.8)	<0.0001
**B2M (continuous.) log**	1.4 (1.3;1.5)	<0.0001	1.3 (1.2;1.3)	<0.0001
**Albumin (>=3.5, < 3.5)**	1.5 (1.4;1.7)	<0.0001	1.5 (1.3; 1.6)	<0.0001
**Albumin (cont.)**	0.7 (0.6;0.7)	<0.0001	0.7 (0.7; 0.8)	<0.0001
**ISS**		<0.0001		<0.0001
**I**	1		1 (ref)	
**II**	1.8 (1.5; 2.0)	<0.0001	1.4 (1.3; 1.6)	<0.0001

**III**	2.6 (2.2; 3.0)	<0.0001	1.9 (1.7; 2.2)	<0.0001
**Creatinine (<180, >=180)**	1.6 (1.5;1.8)	<0.0001	1.4 (1.3; 1.6)	<0.0001
**Creatinine (cont, log)**	1.3 (1.2;1.3)	<0.0001	1.1 (1.1; 1.2)	<0.0001
**LDH (above normal)**	1.4 (1.2;1.5)	<0.0001	1.3 (1.1; 1.4)	<0.0001
**LDH (cont., log)**	1.6 (1.4;1.7)	<0.0001	1.3 (1.2; 1.4)	<0.0001

Ref = Reference. BMPC% = Bone marrow plasma cell %. Cont. = continuous value. Immunoparesis = one or more of uninvolved immunoglobulins below the lower normal levels IgG < 6.1 g/L, IgA < 0.70 g/L and/or IgM <0.39g/L. Immunoparesis 1 vs. 2 Ig = 1 or 2 uninvolved immunoglobulins below lower normal level. B2M = beta-2-microglobulin, ISS = International Staging System. LDH = Lactate dehydrogenase.
