# Supplementary material for: Immunoparesis in newly diagnosed Multiple Myeloma patients: Effects on overall survival and progression free survival in the Danish population
Source: PLoS One. 2017 Dec 7;12(12):e0188988. doi: 10.1371/journal.pone.0188988 (PMC5720701; doi:10.1371/journal.pone.0188988)
Supplement: S2 Table — (PDF) [file pone.0188988.s002.pdf]

S2 Table: Multivariable analysis of the effect of immunoparesis in calendar periods 2005-2008 and 2009-2013 divided by age groups.

≤ 65	OS HR (95% CI)	OS P value	PFS HR (95% CI)	PFS P value
**2005-2008 (n=331)**				
**Immunoparesis**	0.7 (0.4;1.2)	0.18	1.2 (0.8; 1.8)	0.49
**Immunoparesis 25% reduction**	1.1 (0.7;1.6)	0.73	1.3 (0.9; 1.8)	0.15
**2009-2013 (n=414)**				
**Immunoparesis**	0.6 (0.4; 0.9)	0.026	1.2 (0.7; 1.9)	0.52
**Immunoparesis 25% reduction**	0.7 (0.5; 1.1)	0.11	1.1 (0.8; 1.6)	0.55
**> 65**	**OS HR (95% CI)**	**OS P value**	**PFS HR (95% CI)**	**PFS P value**
**2005-2008 (n=473)**				
**Immunoparesis**	1.1 (0.8; 1.5)	0.47	1.3 (0.9; 1.8)	0.13
**Immunoparesis 25% reduction**	1.2 (0.9; 1.5)	0.23	1.4 (1.1; 1.9)	0.019
**2009-2013 (n=769)**				
**Immunoparesis**	0.9 (0.6; 1.2)	0.35	1.1 (0.8; 1.6)	0.43
**Immunoparesis 25% reduction**	1.1 (0.8; 1.4)	0.56	1.4 (1.1; 1.8)	0.007

Immunoparesis = one or more of uninvolved immunoglobulins below the lower nomal levels IgG < 6.1 g/L, IgA < 0.70 g/L and/or IgM <0.39g/L. Immunoparesis 25% = at least one uninvolved immunoglobulin with a 25% reduction from lower normal level. All hazard ratios (HR) and corresponding p-values are adjusted for all significant risk factors for OS and PFS as shown in table 2a and 2b.
